# An Integrative Study of *Scrophularia takesimensis* Nakai in an Ovalbumin-Induced Murine Model of Asthma: The Effect on T Helper 2 Cell Activation

**DOI:** 10.3390/pharmaceutics16040529

**Published:** 2024-04-12

**Authors:** Yun-Soo Seo, Jun-Ho Song, Hyo Seon Kim, Hyeon Hwa Nam, Sungyu Yang, Goya Choi, Sung-Wook Chae, Jeongmin Lee, Bokyung Jung, Joong-Sun Kim, Inkyu Park

**Affiliations:** 1 Herbal Medicine Resources Research Center, Korea Institute of Oriental Medicine, Naju 58245, Republic of Korea; sys0109@kiom.re.kr (Y.-S.S.); hs0320@kiom.re.kr (H.S.K.); namhh@kiom.re.kr (H.H.N.); sgyang81@kiom.re.kr (S.Y.); serparas@kiom.re.kr (G.C.); 2 Center for Companion Animal New Drug Development, Jeonbuk Branch, Korea Institute of Toxicology, Jeongeup 56212, Republic of Korea; kendall@kiom.re.kr; 3 Department of Biology, Chungbuk National University, Cheongju 28644, Republic of Korea; jhsong@chungbuk.ac.kr; 4 KM Convergence Research Division, Korea Institute of Oriental Medicine, 1672 Yuseongdae-ro, Yuseong-gu, Daejeon 34054, Republic of Korea; 5 College of Veterinary Medicine and BK21 FOUR Program, Chonnam National University, Gwangju 61186, Republic of Korea; 216932@jnu.ac.kr (J.L.); bkjung@jnu.ac.kr (B.J.); 6 Department of Biology and Chemistry, Changwon National University, Changwon 51140, Republic of Korea

**Keywords:** *Scrophularia takesimensis*, chloroplast genome, phylogenetic analysis, asthma, regulate Th2 cell, airway inflammation

## Abstract

*Scrophularia* have traditionally been used as herbal medicines to treat neuritis, sore throats, and laryngitis. In particular, *S. takesimensis*, a Korean endemic species with restricted distribution on Ulleung Island, holds significant resource and genetic value. However, its pharmacological properties have not been thoroughly evaluated. Thus, we provide detailed morphological characteristics and genomic information for *S. takesimensis* in this study. Moreover, its pharmacological activity was evaluated in an ovalbumin-induced asthma rat model, using extracts of *S. takesimensis* roots (100 or 200 mg/kg). The distinguishing features of *S. takesimensis* from related species include the presence or absence of stem wings, leaf shape, and habitat. The chloroplast (cp) genome of this species is 152,420 bp long and exhibits a conserved quadripartite structure. A total of 114 genes were identified, which included 80 protein-coding genes, 30 transfer RNA (tRNA) genes, and 4 ribosomal RNA (rRNA) genes. The gene order, content, and orientation of the *S. takesimensis* cp genome was highly conserved and consistent with the general structure observed in *S. buergeriana* and *S. ningpoensis* cp genomes. Confirming the anti-inflammatory effects of *S. takesimensis* extract (STE) using an established mouse model of ovalbumin-induced asthma, we observed reduced asthmatic phenotypes, including inflammatory cell infiltration, mucus production, and suppression of T helper 2 (Th2) cell. Furthermore, STE treatment reduced Th2 cell activation and differentiation. This study underscores the medicinal value of *S. takesimensis*. The importance of preserving *S. takesimensis* was revealed and crucial insights were provided for further research on its utilization as a medicinal resource.

## 1. Introduction

The genus *Scrophularia* L., belonging to the family Scrophulariaceae Juss., comprises more than 200 species that are widely distributed in temperate regions of the Northern Hemisphere [1,2,3]. In Eastern Asia, the dried roots of *Scrophularia* species have traditionally been used as herbal medicines to control neuritis, sore throat, and laryngitis [4,5,6]. Previous studies showed that the *Scrophularia* has anti-inflammatory activities and immune-enhancing effects in in vivo models, such as the ovalbumin (OVA)-induced asthma mouse, and in vitro models including the Raw 264.7, MOLT-4, and SH-SY5Y cells [7]. *Scrophularia* is widely used in traditional medicine, but still there was a lack of evidence in clinical and preclinical studies.

In Korea, the *Scrophularia* genus has been recognized as having seven taxa, six species, and one variety, based on morphological characteristics [8]. Among these species, *S. takesimensis* Nakai (Korean: Seom-Hyun-Sam), a Korean endemic species, has a restricted distribution on Ulleung Island. Given the highly limited distribution of *S. takesimensis*, previous studies on its population genetics, conservation status, and phylogenetic position have been carried out [9,10,11,12,13].

Determining chloroplast (cp) genome sequences is helpful for plant taxonomy, species identification, population genetics, phylogenetic analysis, and diversity and evolution studies [14,15]. While several cp genomes within the *Scrophularia* genus have been reported [16,17,18], and despite being a valuable plant, little genomic information is available on *Scrophularia*. 

Several studies have evaluated the biological properties of Asian *Scrophularia* species. They exhibit various biological properties, including neuroprotective and antioxidant activities. These properties have been linked to several active constituents, notably phenylpropanoids, iridoids, and E-p-methoxycinnamic acid [19,20,21]. Additionally, they can suppress the inflammatory response in allergic disease models by regulating cytokine production [4,22]. Our previous study confirmed that *S. buergeriana* Miq. and *S. koraiensis* Nakai effectively reduced airway inflammation in an OVA-induced allergic asthma model [23,24]. 

However, most studies on the biological properties of *Scrophularia* have solely focused on species that are distributed in Asia or listed in their pharmacopoeias. The pharmacological properties of the Korean endemic species, which has high resource and genetic value, remain to be evaluated. 

Thus, in this integrative study, we describe the morphological and genomic characteristics of Korean endemic *S. takesimensis*. We also examined the effects of *S. takesimensis* on OVA-induced allergic airway inflammation.

## 2. Materials and Methods

### 2.1. Plant Materials

The plant material, *S. takesimensis*, was obtained from the Department of Herbal Crop Research (Eumseong-gun, Chungcheongbuk-do, Republic of Korea), originally collected from its natural habitats (Buk-myeon, Ulleung-gun, and Gyeongsangbuk-do). Accurate identification relied on protologues and other relevant taxonomic studies. A voucher specimen (2-18-0146) has been deposited in the Korean Herbarium of Standard Herbal Resources (Index Herbariorum code: KIOM) at the Korea Institute of Oriental Medicine (Naju, Republic of Korea).

### 2.2. Morphological Observations

The field photographs were captured using a digital camera. Major morphological characteristics were measured using digital Vernier calipers (CD-20AX; Mitutoyo, Sakado, Japan). The leaves and flowers were examined and photographed using a stereomicroscope (SZX16; Olympus, Tokyo, Japan).

### 2.3. Genome Sequencing and Assembly

The total deoxyribo nucleic acid (DNA) of the tested species was extracted using a modified Cetrimonium bromide (CTAB) method [25]. The library was prepared from total genomic DNA using the TruSeq DNA Nano kit (TruSeq; Illumina, San Diego, CA, USA), following the manufacturer’s protocols. Short-insert paired-end sequencing libraries were established and sequenced using the NextSeq500 platform (Illumina, San Diego, CA, USA). As a result, trimmed reads (Phred quality score ≥ 20) were assembled using the CLC genome assembler (ver. 4.2.1; CLC Inc., Aarhus, Denmark) with the default parameters. Based on the aligned paired-end reads, SOAPdenovo gap closure was used to fill the gaps in the sequences [26]. The contigs were aligned to the National Center for Biotechnology Information non-redundant databased (NCBI nrDB) for the identification of cp contigs, and Nucmer was employed to extract these contigs from the overall contig dataset [27]. The contigs were arranged based on the reference cp genome sequence of *S. takesimensis* (NC026202). The new cp genome sequences of *S. takesimensis* obtained in this study were deposited in the NCBI GenBank database under accession number OQ580987.

### 2.4. Genome Annotation and Comparative Analysis

Gene annotation of *S. takesimensis* cp genome was performed using the GeSeq [28]. The protein-coding sequences underwent manual curation, were validated using Artemis [29], and were cross-verified against the NCBI protein database. tRNAs were validated using tRNAscan SE 1.21 [30]. The inverted repeat (IR) region sequences were confirmed utilizing an IR finder and RepEx [31]. Circular maps of the *S. takesimensis* cp genome were generated using OGDRAW [32]. The mVISTA program was employed in Shuffle-LAGAN mode to conduct a comparative analysis of the cp genomes with *S. takesimensis* as the reference [33]. Nucleotide variability (Pi) calculations were performed using DnaSP ver. 6 [34]. To ascertain precise genetic variants, the coding sequences (CDSs), introns, and intergenic spacer (IGS) regions were analyzed individually. Simple sequence repeats (SSRs) were identified using MISA ver. 2.1 [35], where the minimum number of repeat parameters was set to ten, five, four, three, three, and three for mono-, di-, tri-, tetra-, penta-, and hexanucleotides, respectively. Tandem repeats with a length of ≥ 20 bp were detected using the Tandem Repeats Finder [36], applying a minimum alignment score of 50 and a maximum period size of 500; the identity of repeats was set to ≥ 90%.

### 2.5. Phylogenetic Analysis

For phylogenetic analysis, a total of 11 cp genomes, including nine from *Scrophularia* samples, and *Verbascum chinense* (L.) *Santapau* and *Verbascum phoeniceum* L. as outgroups, were utilized. Among these, eight cp genome sequences were obtained from the NCBI GenBank database (Supplementary Table S1). Two matrices encompassing 78 conserved protein-coding genes (CDSs) were employed. Using MAFFT ver. 7.388 [37], the CDS sets underwent alignment. Subsequently, each aligned gene was extracted using Geneious software ver. 2023.2.1 (https://www.geneious.com; accessed on 1 January 2023.), and the genes were organized alphabetically. The concatenated gene dataset was constructed using Geneious software ver. 2023.2.1 and the CDS dataset. GBlocks ver. 5 [38] was applied to filter the alignment datasets and eliminate ambiguously aligned regions. The best-fitting model for nucleotide substitutions was determined through the Akaike information criterion in jModelTest V2.1.10 [39], selecting the generalised time reversible + gamma distributed with invariant sites (GTR+G+I) model for maximum likelihood (ML) analysis and the GTR+G model for Bayesian inference (BI) analysis. ML analysis was executed using RaxML v. 8.0.5 [40] with 1000 bootstrap replicates, while BI analysis utilized MrBayes 3.2.2 [41], involving two independent runs of four simultaneous chains for 5,000,000 generations via the Markov chain Monte Carlo algorithm. Trees were sampled every 5000 generations, discarding the first 25% as burn-ins. The trees were inferred using a 50% majority-rule consensus to estimate posterior probabilities, and the reconstructed trees were visualized using Figtree V.1.4.2 [42].

### 2.6. Preparation of S. takesimensis Extract (STE)

The dried and ground roots of *S. takesimensis* (92.5 g) were refluxed in 70% EtOH at 95 °C for 2 h. The extract, obtained by filtering through filter paper and concentrating in vacuum, weighed 53.91 g (58.28% of yield, *w*/*w*), which was stored at 4 °C for this study.

### 2.7. Animals

Seven-week-old female BALB/c mice (20~22 g) were acquired from Dooyeol Biotech (Seoul, Republic of Korea). The animals were maintained in a room under controlled conditions (temperature, 23 °C; relative humidity, 50%; lit electrically from 08:00–20:00; 13–18 air changes per hour). Six mice were in each cage. The mice were subjected to a week-long quarantine and acclimation period. They were housed under standard conditions, with full access to tap water and food. They were divided into the following four groups of six mice each: saline control (normal control, NC), OVA-induced asthma group (OVA, vehicle treatment), and OVA with 100 or 200 mg/kg STE by oral administration (STE100 and STE200, respectively). Moreover, dexamethasone (DEX) was used in a positive control group that was injected as previously described [23,24]. The study was approved by the Institutional Ethics Committee of Chonnam National University (CNU IACUC-YB-2022-33). All experimental design procedures are indicated in Figure 6A.

### 2.8. Experimental Procedures

Asthma was induced in mice using OVA (Sigma-Aldrich, St Louis, MO, USA), as previously described [43]. On days 0 and 7, the mice received a brief sensitization through intraperitoneal injection of 200 μL OVA/aluminum hydroxide (50 g). On days 14, 15, 16, and 17 after the last sensitization, 25 g (50 µL) OVA was administered intranasally. Between days 11 and 17, STE (100 or 200 mg/kg) was administered once daily. On day 18, BALF from each mouse was obtained by lavage using 1 mL of Dulbecco’s phosphate-buffered saline (PBS; Gibco, New York, NY, USA) after euthanizing by intravenous injection of a combination of alfaxalone (85 mg/kg) and xylazine (10 mg/kg). Cells from BALF samples (100 μL) were placed on a slide through a cytospin and stained with Diff-Quik^®^ reagent (Sysmex Co., Seoul, Republic of Korea) to evaluate differential cell counts.

### 2.9. Histology

Lung tissue samples were collected and fixed in neutral buffered formalin (10% *v*/*v*) overnight. After fixing, the tissue was embedded in paraffin and sectioned to a thickness of 4 μm for histological analysis. Sections were stained with hematoxylin and eosin (H&E) solution (Sigma-Aldrich) or periodic acid–Schiff (PAS) solution (IMEB Inc., San Marcos, TX, USA) to assess histological alterations and mucus production. Tissue slides were imaged with a panoramic DESK slide scanner (3DHISTECH, Budapest, Hungary). Each tissue section was scored on a scale of zero to four according to the extent of interstitial inflammation, alveolar wall thickening, peribronchial inflammation, and interstitial edema (zero ≤ 10%, one = up to 30%, two = up to 50%, three = up to 70%, four ≥ 70%) [44].

### 2.10. Measurement of T Helper 2 (Th2) Cytokine and Immunoglobulin E (IgE) Levels in Bronchoalveolar Lavage Fluid (BALF)

Ineterleukin-4 (IL-4) levels in the BALF were measured using enzyme-linked immunosorbent assay (ELISA) kits (Mouse IL-4 DuoSet ELISA, DY404-05; R&D Systems, Minneapolis, MN, USA), following the manufacturer’s instructions. Total and OVA-specific IgE in the BALF were quantified using ELISA kits (Mouse IgE ELISA Kit, ab157718, Abcam, Cambridge, UK; LEGEND MAX™ Mouse OVA Specific IgE ELISA Kit, BioLegend, San Diego, CA, USA) [45].

### 2.11. Flow Cytometry

Cells extracted from the lungs were stained with fluorescein isothiocyanate (FITC)-conjugated anti-cluster of differentiation (CD) 4, Alexa Fluor 700-conjugated anti-CD3, and PE-conjugated anti-CD25 antibodies to examine activated T cells, as previously described [46]. The number of activated T cells and Th2 cells were obtained by multiplying their frequencies by CD3+ cells. Antibodies were purchased from the following three different suppliers: BD Biosciences, eBioscience, and BioLegend. FlowJo software version 10.6 (TreeStar, Ashland, OR, USA) and an LSR Fortessa X-20 flow cytometer (BD Biosciences, Franklin Lakes, NJ, USA) were used to evaluate the labelled cells.

### 2.12. Statistical Analysis

GraphPad Prism (version 9.3.1, GraphPad Software, San Diego, CA, USA) was used for statistical analysis. A one-way analysis of variance (ANOVA), followed by a Dunnett’s test, was used to determine the statistical significance of the data. Means and standard deviations were used to express the results. Statistical significance was set at *p* < 0.05.

## 3. Results

### 3.1. Description and Morphological Characteristics of Scrophularia Takesimensis

**Figure 1:**
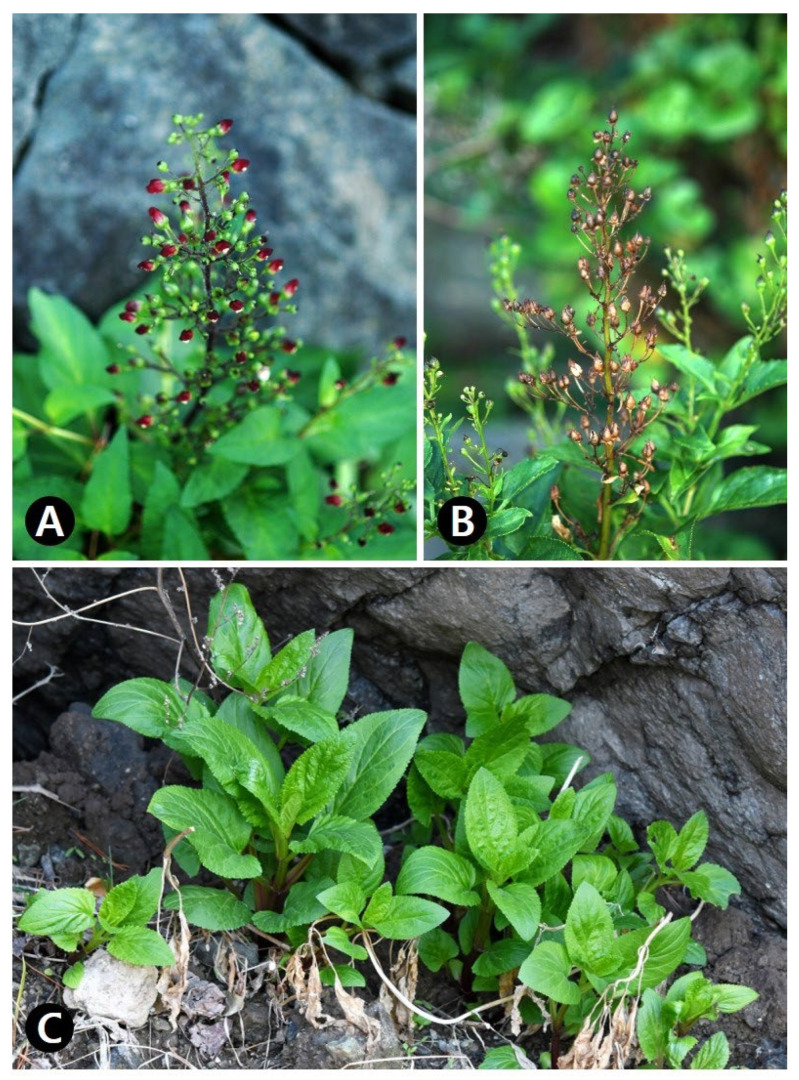
Habitat and morphology of Korean endemic *Scrophularia takesimensis* in natural population of Ulleungdo. (**A**) Inflorescence. (**B**) Infructescence. (**C**) Habitat and previous flowering stage.

Herbs perennial, 0.7−1.5 m tall. Root taproot, simple, and stout. Stems ascending, solitary to several, 0.5−1.5 cm wide, grooved and not winged, glabrous. Leaves opposite; petiole 2.5−6 cm long, winged, partly clasping stem at base, glabrous; blade ovate, 10−20 × 6−15 cm, apex acute or rarely obtuse, base cuneate to rounded, margins serrate almost without spinose tooth, both surfaces glabrous. Inflorescences terminal and axillary, panicle-like cymes, many-flowered with glandular hairs; bracts lanceolate to linear, 1−3 cm long; pedicels 1−2 cm long. Flowers flowering from June to Aug, calyx campanulate, 5-lobed; calyx lobes ovate, apex rounded; corolla 2-lipped, red to reddish purple, urceolate; upper lip 2-lobed, lobes of upper lip orbicular; lower lip 3-lobed, middle lobe reflexed; stamens 4, didynamous; filaments filiform; anthers yellow; ovary broadly ovoid, not submerged in nectary disk; style filiform. Fruits capsules fruiting Aug to Sep, ovoid, 0.5−1.5 × 0.5−1 cm. Seeds brown to dark brown, ellipsoidal to ovoid (Figure 1).

*S. takesimensis* thrives in a distinct habitat (seashore), unlike the other two species (forest) listed in the Korean Herbal Pharmacopoeia. Moreover, it is distinguished from related species by its simple and stout root, red corolla, and serrate leaves, with minimal spinose teeth (Table 1).

### 3.2. Chloroplast Genome Organization and Repeat Sequence of S. takesimensis

**Figure 2:**
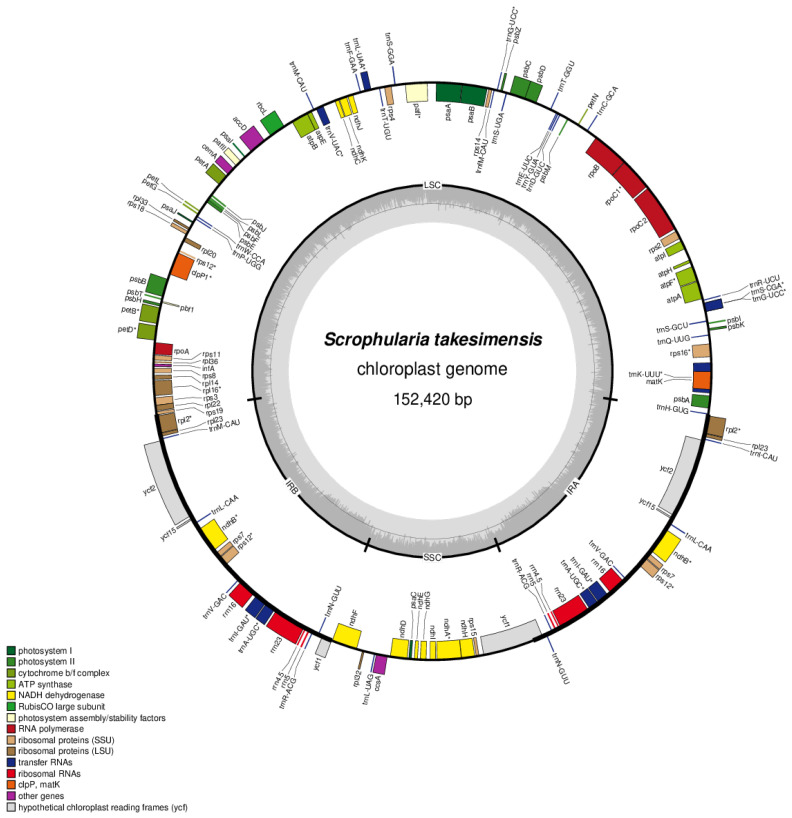
Circular gene map of the chloroplast genome from *Scrophularia takesimensis*. Genes drawn inside the circle are transcribed clockwise, and those outside the circle are transcribed counterclockwise. The darker gray in the inner circle represents GC content. * means genes containing introns.

*S. takesimensis* was sequenced at approximately 8000× coverage, resulting in the generation of 34 Gb of raw paired-end reads and 32 Gb of trimmed reads (refer to Table S1). The entire circular chloroplast (cp) genome measured 152,420 bp in length, exhibiting the typical quadripartite structure of cp genomes. This structure comprised a pair of inverted repeats (IRs; 25,475 bp) separated by the large single copy (LSC; 83,526 bp) and short single copy (SSC; 17,938 bp) regions (see Figure 2 and Table 2). The complete cp genomes were of high quality (Supplementary Table S2). The overall guanine-cytosine (GC) content was approximately 38%, and slightly higher in the IR region (43%) than in the LSC (36%) or SSC (32%) regions. These four cp genomes comprised 114 genes, including 80 protein-coding, 4 rRNA, and 30 tRNA genes (Table S3). They had 18 intron-containing genes, 15 of which had a single intron, and 2 of which (*ycf3* and *clpP*) had three introns with duplicate genes (*ndhB*, *trnI-GAU*, and *trnA-UGC*) in the IR regions (Table S4).

### 3.3. Comprehensive Comparative Analysis of Scrophularia Chloroplast Genomes

To identify repetitive sequences in the cp genomes, we studied simple sequence repeats (SSRs) and tandem repeats in the three *Scrophularia* cp genomes. The mononucleotide motif was the most abundant of all the cp genomes studied, followed by dinucleotide motif repeats (Figure S2A). The *Scrophularia* cp genomes exhibited repeat sequences in intergenic spacer (IGS) regions, with 34–38 SSRs (Figure S2B). Most tandem repeats, predominantly 21–40 bp long, were located in the LSC and SSC of the IGS region (Figure S2C,D). We also identified one–four tandem repeats of >71 bp in *Scrophularia*.

**Figure 3:**
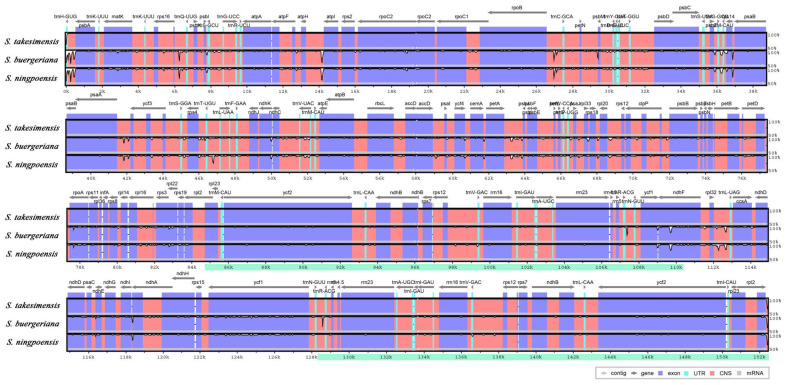
Comparison of *Scrophularia* cp genomes using mVISTA. The complete cp genomes of three *Scrophularia* species were compared, with *S. takesimensis* as a reference. Blue block: conserved genes, sky-blue block: transfer RNA (tRNA) and rRNA, red block: CNS. White represents the regions with sequence variation among the three *Scrophularia*. CNS: conserved non-coding sequences; cp: chloroplast; rRNA: ribosomal RNA; tRNA: transfer RNA.

Sequence identities were analyzed using the mVISTA program with the *S. takesimensis* cp genome as a reference, revealing a well-conserved overall structure among the *Scrophularia* cp genomes (Figure 3). As expected, the genic regions were more conserved than the IGS regions, with the highest divergences observed in the LSC and SSC regions. 

**Figure 4:**
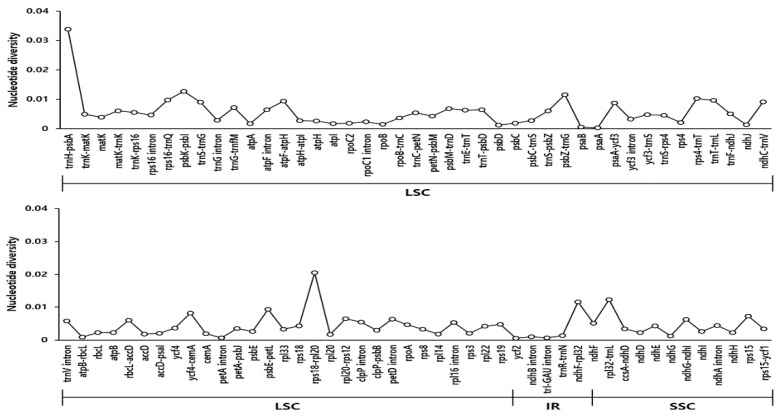
Comparison of the nucleotide diversity (Pi) values among the *Scrophularia*. Pi value in *Scrophularia* chloroplast genomes and excludes regions with Pi = 0. LSC: Large single copy; IR: inverted repeat; SSC: small single copy.

We conducted an analysis of genetic divergence utilizing three *Scrophularia* cp genomes and identified species-specific mutations through pairwise alignment criteria in *S. takesimensis* (Figure 4). Nucleotide diversity (Pi) values were determined for the three *Scrophularia* cp genomes, along with species-specific Pi values. Overall, the *Scrophularia* cp genome exhibited an average Pi value of 0.005. Divergent regions were observed in the non-coding trnH-psbA, rps18-rpl20 in the large single copy (LSC), and ndhF-rpl32 and rpl32-trnL in the short single copy (SSC). Notably, trnH-psbA displayed the highest Pi value of approximately 0.02, followed by trnH-psbA (0.033) in the LSC region. Overall, our findings indicated that the cp genomes of *S. takesimensis* were highly conserved in terms of genome structure and gene order compared to those of other *Scrophularia* species.

### 3.4. Phylogenetic Relationships among Scrophularia Genus

**Figure 5:**
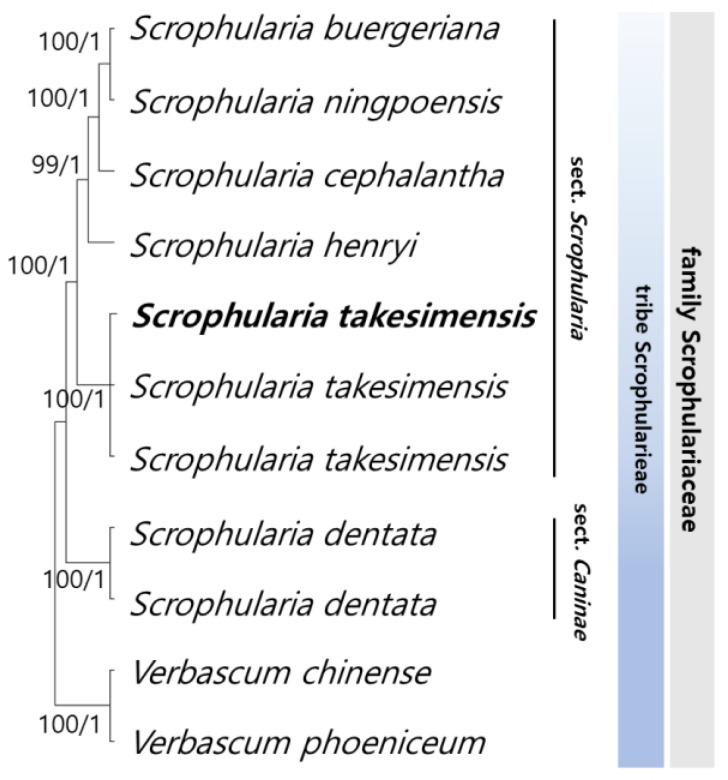
A phylogenetic tree of *Scrophularia* representing the maximum likelihood (ML) bootstrap and Bayesian inference (BI) probability. The number of branches above or below represents the ML bootstrap and BI probability support values. The cp genomes completed in this study are indicated with bold text. BI: Bayesian inference; ML: maximum likelihood; PP: posterior probabilities.

To elucidate the phylogenetic relationships within *Scrophularia*, we identified 78 conserved protein-coding sequences by aligning 69,846 bp shared among all the *Scrophularia* samples, with *Verbascum chinense* and *V. phoeniceum* serving as outgroups (Figure 5). The maximum likelihood (ML) and Bayesian inference (BI) topologies exhibited remarkable congruence with both the whole genome sequences and coding sequences (CDS) datasets. The phylogenetic analyses provided robust support for all lineages except one (ML = 100%, BI = 1.0). Notably, the six *Scrophularia* species within the tribe formed a well-clustered group. *S. buergeriana* and *S. ningpoensis* Hemsl. constituted a monophyletic group, with *S. cephalantha* Nakai identified as a sister group. Overall, clustering revealed a close relationship between *S. buergeriana* and *S. ningpoensis*, with *S. takesimensis* as their sister group, while *S. dentata* Royle ex Benth. showed a distinct phylogenetic position within *Scrophularia*.

### 3.5. Effects of S. takesimensis Extract (STE) on Lung Histopathological Change and Number of Inflammatory Cells in the Bronchoalveolar Lavage Fluid (BALF) of an Ovalbumin (OVA)-Induced Asthmatic Animal Model

**Figure 6:**
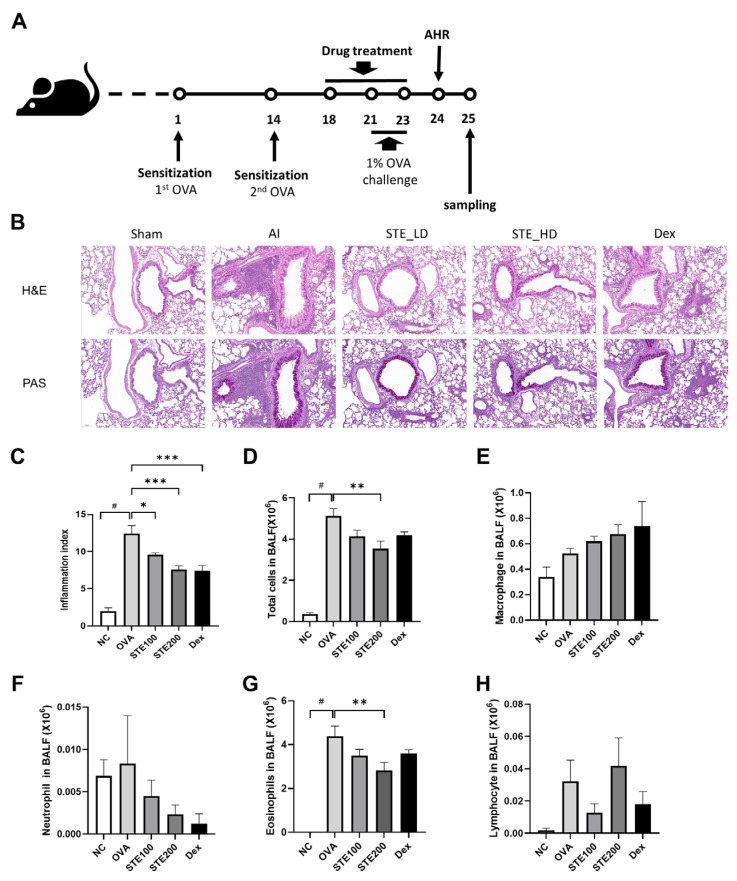
Ovalbumin (OVA)-induced airway inflammation and mucus production were reduced by *S. takesimensis* extract (STE). Schematics diagram of asthma-induced mice model (**A**). Lung sections were stained using hematoxylin and eosin (H&E) and periodic acid–Schiff (PAS) stain (magnification 200×) (**B**). Cells were separated using centrifugation in bronchoalveolar lavage fluid (BALF) and stained with Diff-Quick stain reagent. The inflammation index (**C**), total cells (**D**), macrophages (**E**), neutrophils (**F**), eosinophils (**G**), and lymphocytes (**H**) in BALF are exhibited (*n* = 6). NC, normal control; OVA, group sensitized/challenged with OVA; STE100, OVA + 100 mg/kg of STE; STE200, OVA + 200 mg/kg of STE; Dex, positive control., ^#^ *p* < 0.05 compared with the NC group, * *p* < 0.05, ** *p* < 0.01, *** *p* < 0.001 compared with the OVA group.

H&E and PAS staining were performed to investigate the changes in lung tissue (Figure 6B). The results demonstrated that the OVA-induced asthmatic group exhibited a higher accumulation of immune cells than the control group. However, STE-treated and Dex-treated groups, which is the positive control group, showed a dose-dependent decrease in immune cell count, compared to that in the OVA-induced asthmatic group (Figure 6C,D).

PAS staining was used to measure mucus hypersecretion. In this study, we confirmed a significant increase in mucus production in the OVA-induced asthmatic group compared with the control group, and a decrease in mucus production was observed in the STE-treated and DEX-treated groups, compared with that in the OVA-induced asthmatic group. The STE-treated group exhibited a dose-dependent reduction in mucus production (Figure 6B). 

A significant increase in the total number of cells and eosinophils was detected in the OVA-induced asthmatic group compared with the control group (*p* < 0.05). The STE200-treated group showed a significantly reduced number of total cells, compared with the OVA-induced asthmatic model (Figure 6D–H). 

### 3.6. Effects of S. takesimensis Extract (STE) on Interleukin-4 (IL-4) and Immunoglobulin E (IgE) Levels in Bronchoalveolar Lavage Fluid (BALF) of Asthmatic Animal Model

**Figure 7:**
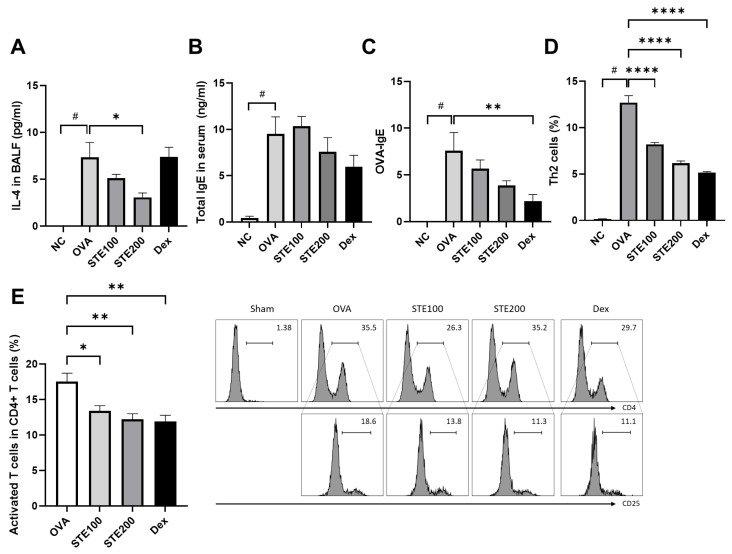
*S. takesimensis* Extract (STE) effectively reduces pro-inflammatory cytokines interleukin-4 (IL-4) and immunoglobulin E (IgE), as well as T cell activation, in asthmatic mice. Enzyme-linked immunosorbent assay (ELISA) was used to analyze the BALF collected from the mice, revealing IL-4 levels (**A**) and antigen-specific IgE (**B**,**C**) in bronchoalveolar lavage fluid (BALF). The cells were analyzed using flow cytometry. T helper 2 (Th2) cells (**D**) and activated T cells (**E**) data are shown (*n* = 6). NC, normal control; OVA, OVA-sensitized/challenged group; STE100, OVA + 100 mg/kg STE; STE200, OVA + 200 mg/kg STE; Dex, positive control. # *p* < 0.05 compared with the NC group, * *p* < 0.05, ** *p* < 0.01, **** *p* < 0.0001 compared with the OVA group compared with the OVA group.

IL-4 cytokine levels were measured in the BALF and showed a significant increase in the OVA-induced asthma group, compared with the control group. Decreased IL-4 levels were detected in the STE-treated group compared with those in the OVA-induced asthma group, showcasing a particularly significant decline in the STE200 group (Figure 7A). However, the Dex-treated group was no different from the OVA-induced asthma group with regard to IL-4 levels. A significant increase in IgE production was observed in the OVA-induced asthma group, when compared with the control group (Figure 7B,C). While STE treatment influenced OVA-specific IgE levels, only the STE200-treated and Dex-treated groups demonstrated component-dependent decreased levels of OVA-specific IgE compared with the OVA-induced asthma group (Figure 7C), although there was no significant difference.

### 3.7. Effects of S. takesimensis Extract (STE) on CD4+ T Cell (Helper T Cell) Activation in the Lung Tissues of Asthmatic Animal Model

T cells were activated in the OVA-induced asthma model, and STE and Dex treatment significantly deactivated T cells, in comparison with the OVA-induced asthma group (Figure 7E). An increase in the number of T helper 2 (Th2) cells was also observed in the OVA-induced asthma group. STE and Dex treatment significantly decreased the percentage of Th2 cells, compared with that in the OVA-induced asthma model (Figure 7D).

## 4. Discussion

*Scrophularia* species have been integral to traditional and folk medicine for centuries with regard to addressing neuritis, sore throats, and laryngitis [4,5,6]. *Scrophularia takesimensis*, an endemic species that shows significant similarities to the medicinal plant *S. buergeriana*, has been well characterized in terms of its morphological traits and anti-inflammatory and antioxidant properties [4,19,20,21,22].

Although the presence or absence of stem wings, leaf shape, and habitat distinguished *S. takesimensis* from other species in this genus, all *Scrophularia* species clustered into monophyletic groups. Genetically, *S. takesimensis* was more closely related to *S. buergeriana*, *S. ningpoensis*, *S. cephalantha*, and *S. henryi* Hemsl. from the same section (*Scrophularia* sect. *Scrophularia*). According to the Korean Herbal Pharmacopoeia, the dried roots *S. buergeriana* and *S. ningpoensis* are used as herbal medicines, known as Scrophulariae Radix [5,6]. This demonstrates that, while *S. takesimensis* is closely related to *S. buergeriana* and *S. ningpoensis* as listed medicines, it possesses distinct features. Nevertheless, *S. takesimensis* has significantly similar properties to treat allergic inflammation akin to *S. buergeriana*.

The phylogeny of medicinal plants has proven to be a useful predictive tool for a more targeted approach [47]. Thus, we expect that the two species, *S. cephalantha* and *S. henryi* clustered in the same clade as *S. takesimensis* of monophyly, would have a similar effect. We suggest an analysis of the inflammatory responses of *S. cephalantha* and *S. henryi* to clarify the evolutionary prediction of medicinal properties in the genus *Scrophularia* for future phylogeny-guided drug discovery.

Several studies have reported the anti-inflammatory, neuroinflammatory, antioxidant, and hepatoprotective effects of *Scrophularia* plant extracts containing iridoid glycosides [20,24,48,49,50]. Harpagoside, an iridoid glycoside, is a major bioactive component in the roots of *Harpagophytum procumbens* (Burch.) DC. ex Meisn. (family Pedaliaceae), which is used when treating chronic rheumatism, osteoarthritis, and arthritis, and is an active constituent of the *Scrophularia* species [51,52,53,54]. 

Allergic asthma is a chronic respiratory disease caused by an impaired immune response. Owing to the recent spread of the coronavirus disease 2019, interest in the treatment of pulmonary disorders has rapidly increased [55]. The dried roots of *S. buergeriana* have traditionally been used as herbal medicines to treat fever, edema, neuritis, and laryngitis [4]. Similarly, *S. koraiensis* has protective effects on OVA-induced allergic airway inflammation by suppressing NF-κB phosphorylation and enhancing the Nrf2/HO-1 signaling pathway [23]. 

In this study, STE reduced eosinophilia and pro-inflammatory cytokine production, decreased IgE levels, and alleviated OVA-induced allergic airway inflammation, as supported by histological evidence of reduced airway inflammation and mucus secretion. These results suggest that STE may prove effective for suppressing the development of asthma. In the present study, we evaluated Th2 cell activation to further understand the effects of STE on Th2 cytokine regulation. STE significantly reduced the proportion of activated CD4+ T cells and Th2 cell differentiation under polarization conditions. These results suggest that STE attenuates the reduction in Th2 cytokine-mediated asthmatic responses by directly regulating the activation and differentiation of Th2 cells [46]. Our results also indicate that STE has an efficacy similar to that of *S. buergeriana*, demonstrating the possibility of its incorporation into medicinal materials. Moreover, we underscored the possibility of using morphological and phylogenetic approaches as predictive tools for exploring new medicinal plants.

## 5. Conclusions

This study provides comprehensive data on the morphological, genomic, and biological activities of the endemic plant *S. takesimensis*. This endemic species also has efficacy against asthma, including asthmatic phenotypes, encompassing inflammatory cell infiltration, mucus production, and suppression of Th2 cytokines similar to *S. buergeriana*, which is a closely related species, demonstrating its potential for incorporation into medicinal materials. This integrative study presents the possibility of using morphological and phylogenetic approaches as useful predictive tools for exploring new medicinal plants. Consequently, this study provides insights for screening alternative medicines using predictive values from natural classification and for evaluating the biological properties of endemic species. 

## Figures and Tables

**Table 1:** Major morphological characteristics of the Korean endemic *Scrophularia takesimensis* and related species, *S. buergeriana* and *S. ningpoensis*.

Species	*S. takesimensis*	*S. buergeriana*	*S. ningpoensis*
Habitat	Seashore	Forest	Forest
Height	~1.5 m	~1.8 m	~1.5 m
Root	Simple and stout	Fusiform to conical	Fusiform to conical
Stem	Wingless, glabrous	Wingless, glabrous	Slightly winged, mostly white crisped hairy
Leaf shape	Ovate	Ovate	Mostly ovate
Leaf apex	Acute	Acute	Acute
Leaf margins	Serrate almost without spinose tooth	Serrate with spinose tooth	Serrate with spinose tooth
Leaf base	Cuneate to rounded	Cuneate to rounded	Cuneate to rounded
Inflorescence shape	Panicle-like cymes	Spike-like cymes	Panicle-like cymes
Inflorescence position	Terminal and axillary	Mostly terminal	Terminal and axillary
Flower color	Red	Yellowish green	Red

**Table 2:** Features of *Scrophularia takesimensis* and related species’ chloroplast genomes.

Species	*S. takesimensis*	*S. buergeriana*	*S. ningpoensis*
Accession number	OQ580987	NC_031437	NC_053823
Total cp genome size (bp)	152,420	153,631	153,175
Large single copy (LSC) region (bp)	83,526	84,454	84,255
Inverted repeat (IR) region (bp)	25,478	25,624	25,490
Small single copy (SSC) region (bp)	17,938	17,929	17,938
Total number of genes (unique)	114	114	114
Protein-coding gene (unique)	80	80	80
rRNA (unique)	4	4	4
tRNA (unique)	30	30	30
GC content (%)	38	38	38
LSC (%)	36	36	36
IR (%)	43	43	43
SSC (%)	32	32	32

## Data Availability

The complete chloroplast genome of four *Scrophularia takesimensis* in this study was submitted to the NCBI database (https://www.ncbi.nlm.nih.gov/; accessed on 10 April 2023) with GenBank accession number OQ580987. All the cp genomes used in this study can be found in GenBank, and the GenBank accessions are shown in Additional File 1: Table S6. The other cp genomes used in this study were downloaded from NCBI. The datasets generated and/or analyzed in the current study are available upon request from the corresponding author.

## Supplementary Materials

The following supporting information can be downloaded from https://www.mdpi.com/article/10.3390/pharmaceutics16040529/s1. Figure S1: voucher specimens of the three *Scrophularia* species. A. *S. takesimensis* Nakai., B. *S. buergeriana* Miq., C. *S. ningpoensis* Hemsl; Figure S2: The distribution of the repeat sequences in Scrophularia chloroplast genomes. (A) Distribution of single se-quence repeats (SSRs) types. (B) The number of SSRs in genomic regions. (C) Distribution of tandem repeats in ge-nomic regions. (D) Distribution of lengths of the tandem repeats; Table S1: raw and trimmed read data; Table S2: genome assembly information for *S. takesimensis* chloroplast genomes; Table S3: genes in the chloroplast genomes of *S. takesimensis* species; Table S4: genic introns in *S. takesimensis* chloroplast genomes; Table S5: voucher specimen information for floral micromorphology, palynology, and chloroplast genomes, DNA barcode analysis used in this study; Table S6: chloroplast genomes from NCBI used for phylogenetic analysis.
