# Supplementary material for: An Integrative Study of Scrophularia takesimensis Nakai in an Ovalbumin-Induced Murine Model of Asthma: The Effect on T Helper 2 Cell Activation
Source: Pharmaceutics. 2024 Apr 12;16(4):529. doi: 10.3390/pharmaceutics16040529 (PMC11055152; doi:10.3390/pharmaceutics16040529)
Supplement: Supplementary file 1 [file pharmaceutics-16-00529-s001.zip › pharmaceutics-2839044-supplementary.pdf]

Supplementary Material

An Integrative Study of *Scrophularia takesimensis* Nakai in an Ovalbumin-Induced Murine Model of Asthma: The Effect on T Helper 2 Cell Activation

Yun-Soo Seo†, Jun-Ho Song†, Hyo Seon Kim, Hyeon Hwa Nam, Sungyu Yang, Goya Choi, Sung-Wook Chae, Jeongmin Lee, Bokyung Jung, Joong-Sun Kim* and Inkyu Park*

*Correspondence:
Joong-Sun Kim: centraline@jnu.ac.kr and Inkyu Park: pik6885@cwnu.ac.kr

† These authors contributed equally to this work.

Supplementary Figures and Tables

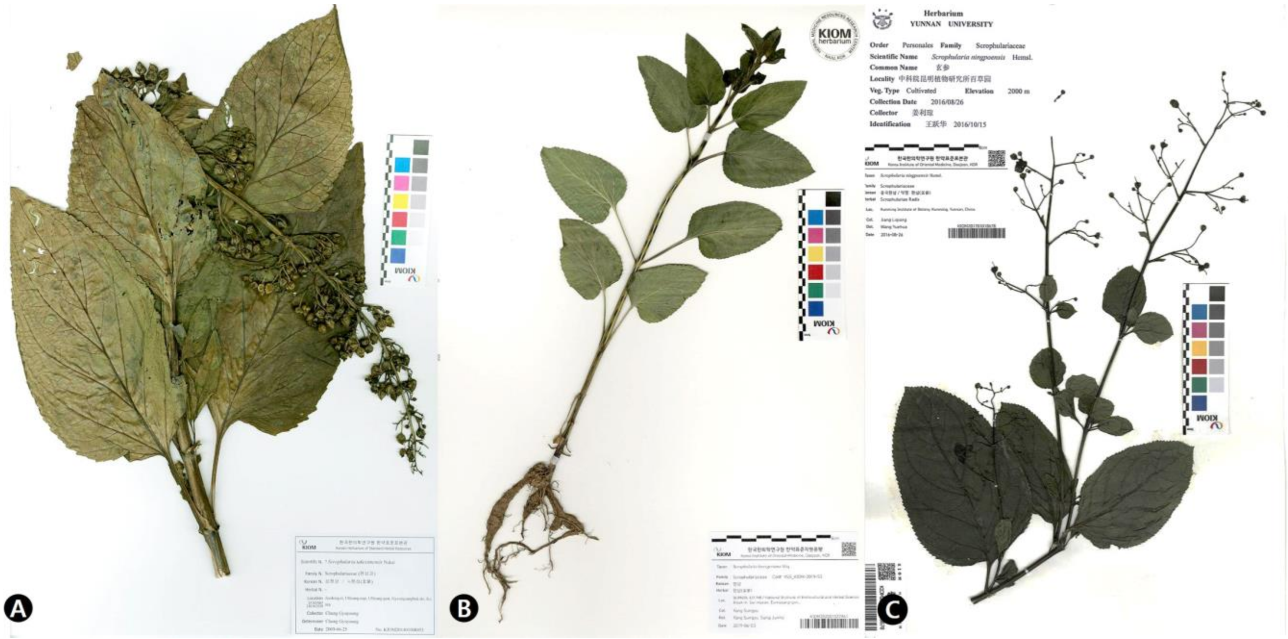

Fig. S1. Voucher specimens of three *Scrophularia* species. A. *S. takesimensis* Nakai. B. *S. buergeriana* Miq. C. *S. ningpoensis* Hemsl.

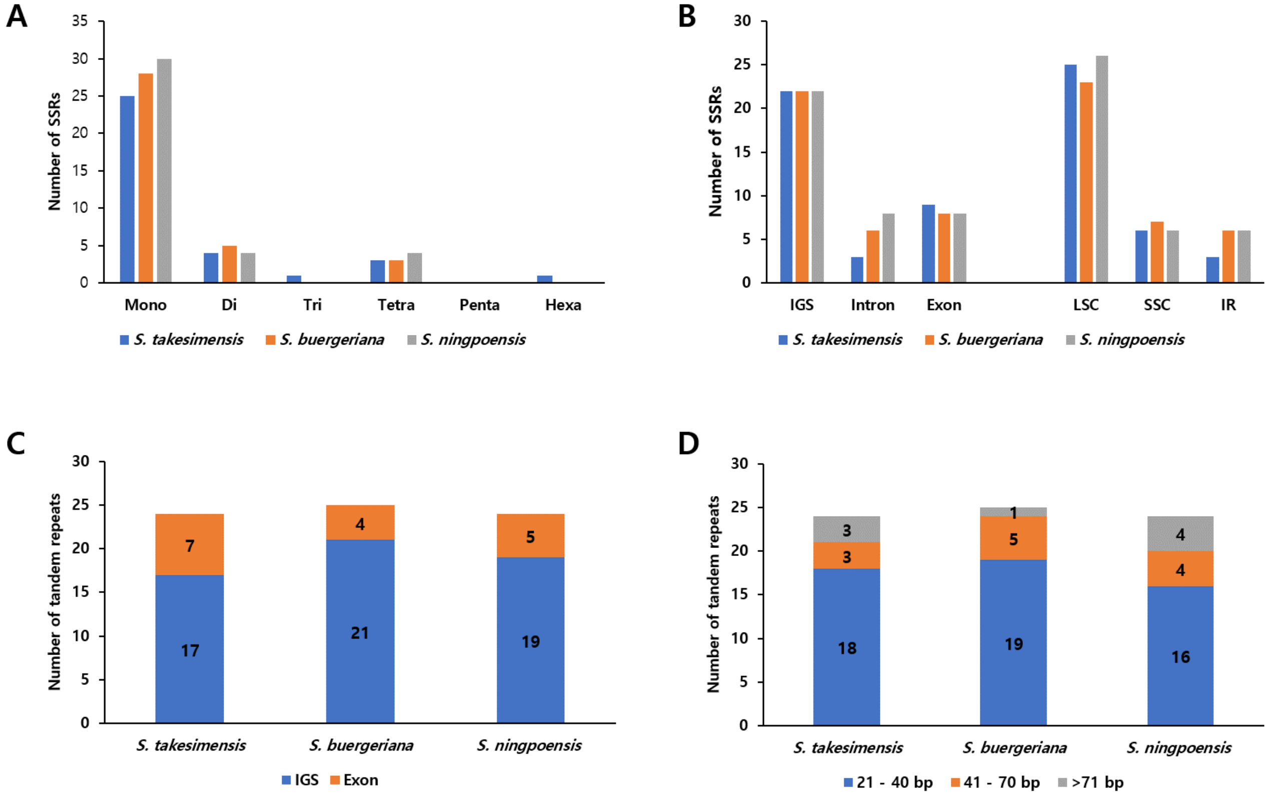

Fig. S2. The distribution of the repeat sequences in *Scrophularia* chloroplast genomes. (A) Distribution of single se-quence repeats (SSRs) types. (B) The number of SSRs in genomic regions. (C) Distribution of tandem repeats in ge-nomic regions. (D) Distribution of lengths of the tandem repeats

Table S1 Raw and trimmed read data

Scientific name	Input reads	Trimmed reads		Total raw bases	Trimmed bases	
S. takesimensis	226,428,024	221,287,500	97.73%	34,190,631,624	32,595,043,159	95.33%

Table S2 Genome assembly information for S. takesimensis chloroplast genomes

Scientific name	Aligned reads (#)	Coverage (x)	Cp genome length (bp)
S. takesimensis	29,525,085	8,000	152,420

Table S3 Genes in the chloroplast genomes of S. takesimensis species

Group of genes	Name of genes
Photosystem I	*psaA, B, C, I, J, ycf3*[2)], *ycf4*
Photosystem II	*psbA, B, C, D, E, F, H, I, J, K, L, M, N, T, Z*
Cytochrome b6/f	*petA, B*[1)], *D*[1)], *G, L, N*
ATP synthase	*atpA, B, E, F*[1)], *H, I*
Rubisco	*rbcL*
NADH oxidoreductase	*ndhA*[1)], *B*[1) 3)], *C, D, E, F, G, H, I, J, K*
Large subunit ribosomal proteins	*rpl2*[1) 3)], *14, 16*[1)], *20, 22, 23*[3)], *32, 33, 36*
Small subunit ribosomal proteins	*rps2, 3, 4, 7*[3)], *8, 11, 12*[2) 3) 4)], *14, 15, 16*[1)], *18, 19*
RNA polymerase	*rpoA, B, C1*[1)], *C2*
Unknown function protein coding gene	*ycf1*[3)], *2*[3)]
Other genes	*accD, ccsA, cemA, clpP*[2)], *matK*
Ribosomal RNAs	*rrn16*[3)], *23*[3)], *4.5*[3)], *5*[3)]
Transfer RNAs	*trnA-UGC*[1)3)], *trnC-GCA, trnD-GUC, trnE-UUC, trnF-GAA, trnfM-CAU, trnG-GCC, trnG-UCC*[1)], *trnH-GUG, trnI-CAU*[3)], *trnI-GAU*[1)3)], *trnK-UUU*[1)], *trnL-CAA*[3)], *trnL-UAA*[1)], *trnL-UAG, trnM-CAU, trnN-GUU*[3)], *trnP-UGG, trnQ-UUG, trnR-ACG*[3)], *trnR-UCU, trnS-GCU, trnS-GGA, trnS-UGA, trnT-GGU, trnT-UGU, trnV-GAC*[3)], *trnV-UAC*[1)], *trnW-CCA, trnY-GUA*

1) Gene containing a single intron, 2) gene containing two introns, 3) two gene copies in IRs, 4) trans-splicing gene

Table S4 Genic introns in S. takesimensis chloroplast genomes

	Gene	Region	exon I	intron I	exon II	intron II	exon III
1	trnk-UUU	LSC	37	37	2509	35	
2	rps16	LSC	39	44	846	223	
3	trnG-UCC	LSC	23	23	684	47	
4	rpoC1	LSC	435	144	722	411	
5	ycf3	LSC	126	435	757	1620	
6	trnL-UAA	LSC	37	128	707	227	726
7	trnV-UAC	LSC	39	37	488	50	
8	rps12	LSC	114	38	584	37	
9	clpP	LSC	69	114		232	
10	petB	LSC	6	69	730	291	634
11	petD	LSC	8	6	722	642	
12	rpl16	LSC	9	8	734	475	
13	rpl2	LSC	393	9	883	399	
14	ndhB	IR	777	393	664	435	
15	trnI-GAU	IR	42	777	679	756	
16	trnA-UGC	IR	38	42	945	35	
17	ndhA	SSC	552	38	813	35	

.

Table S5 Voucher specimen information for floral micromorphology, palynology, and chloroplast genomes, DNA barcode analysis used in this study

No.	Species	Collection information	Coordinates	Collector *collection no.* (Herbarium acronym)
1	*S. takesimensis*	Deogyusan Mt., Seolcheon-myeon, Muju-gun, Jeollabuk-do, Korea	35°51'27.9"N 127°43'18.9"E	B. Choo & Y. Ji *KIOM200901002023* (KIOM)[C, M, P, D]
2		Cheonhwangsan Mt., Icheon-ri, Sangbuk-myeon, Ulju-gun, Ulsan, Korea	35°33'29.4"N 128°58'26.4"E	S.C. Ko *KIOM201601018272* (KIOM)[M, P, D]
3		Baegunsan Mt., Gwangyang-si, Jeollanam-do, Korea	35°03'06.1"N 127°35'31.8"E	Y. Ji & B. Moon *KIOM201101004181* (KIOM)[M, P]

Table S6 Chloroplast genomes from NCBI used for phylogenetic analysis

No.	Tribe	Taxon	GenBank accession number
1	Scrophulariaceae	*Scrophularia takesimensis*	KP718628
2		*Scrophularia dentata*	KT428154
3		*Scrophularia takesimensis*	NC_026202
4		*Scrophularia buergeriana*	NC_031437
5		*Scrophularia dentata*	NC_036942
6		*Scrophularia henryi*	NC_036943
7		*Scrophularia ningpoensis*	NC_053823
8		*Scrophularia cephalantha*	NC_057296
9		*Verbascum phoeniceum*	NC_050920
10		*Verbascum chinense*	NC_051533
